# On Variation in Mindfulness Training: A Multimodal Study of Brief Open Monitoring Meditation on Error Monitoring

**DOI:** 10.3390/brainsci9090226

**Published:** 2019-09-06

**Authors:** Yanli Lin, William D. Eckerle, Ling W. Peng, Jason S. Moser

**Affiliations:** Department of Psychology, Michigan State University, Psychology Building, East Lansing, MI 48823, USA (W.D.E.) (L.W.P.) (J.S.M.)

**Keywords:** mindfulness, error monitoring, ERN, Pe, meditation, open monitoring

## Abstract

A nascent line of research aimed at elucidating the neurocognitive mechanisms of mindfulness has consistently identified a relationship between mindfulness and error monitoring. However, the exact nature of this relationship is unclear, with studies reporting divergent outcomes. The current study sought to clarify the ambiguity by addressing issues related to construct heterogeneity and technical variation in mindfulness training. Specifically, we examined the effects of a brief open monitoring (OM) meditation on neural (error-related negativity (ERN) and error positivity (Pe)) and behavioral indices of error monitoring in one of the largest novice non-meditating samples to date (*N* = 212). Results revealed that the OM meditation enhanced Pe amplitude relative to active controls but did not modulate the ERN or behavioral performance. Moreover, exploratory analyses yielded no relationships between trait mindfulness and the ERN or Pe across either group. Broadly, our findings suggest that technical variation in scope and object of awareness during mindfulness training may differentially modulate the ERN and Pe. Conceptual and methodological implications pertaining to the operationalization of mindfulness and its training are discussed.

## 1. Introduction

For the past two decades, mindfulness, commonly defined as the adoption of a nonelaborative, nonjudgmental awareness to present-moment experience [1,2], has garnered increasing interest for its seemingly innumerable benefits, permeating into the broader social discourse and influencing areas including public health, academia, corporations, and even politics [3,4]. Despite mounting caution from various academic disciplines that enthusiasm for mindfulness may be outpacing scientific progress [5,6,7], the accelerating proliferation and public embracement of mindfulness appear relatively uninterrupted. As with any growing scientific discipline, balancing optimism with rigor represents a formidable and persistent challenge.

In considering the specific influence of science, widespread co-option of mindfulness may be driven by the disproportionate number of studies examining and reporting the effects of mindfulness (i.e., what it does) relative to studies aimed at discerning its underlying mechanisms (i.e., how it works). Research aimed at exploring the salutary effects of mindfulness may be derivative of broader sociocultural interests in self-improvement and social flourishing—motivations that may maintain a collective predilection toward “discovering benefits” [8]. Indeed, this appears reflected in the large and continuously expanding number of clinical, academic, social, and professional interventions from which mindfulness serves as a basis and inspiration. 

Critically, the proliferation of mindfulness-based applications arguably impedes mechanistic investigation—namely, that rapid change in the dissemination and implementation of mindfulness erodes its definition and uniquely challenges methodical operationalization [7,9]. Furthermore, the ever-expanding number of mindfulness-related “benefits” contributes to the intractability in pinpointing general mechanisms that undergird its purported myriad effects. With that said, one potential way to navigate these challenges is to systematically elucidate how mindfulness influences specific well-studied neurocognitive functions that underlie an array of human behaviors.

One such function is error monitoring (also referred to as performance monitoring), a foundational feature of human cognition that facilitates the ability to continuously detect and adjust to errors [10,11,12,13,14]. Importantly, error monitoring is crucial in enabling goal-directed action and promoting behavioral adaptation—core abilities that underlie academic achievement, workplace productivity, mental health, and other outcome variables that are commonly associated with mindfulness. To the extent that the adoption and sustainment of mindfulness constitutes a goal-oriented action [15,16], the very act of being mindful itself—whether through intentional application of state mindfulness toward daily activities (see [17]) or engagement in more formal avenues of mindfulness training such as meditation—is likely to recruit, and possibly modulate, the error monitoring system and its downstream behavioral consequences (e.g., detection of mind wandering and subsequent remedial redirection of attention). Indeed, investigating the nature of the relationship between mindfulness and error monitoring may be promising in understanding the means and extent to which mindfulness exerts its broader influence on contemporary life. 

In contrast to the relative nascency of mindfulness research, error monitoring has been studied extensively for over 50 years (e.g., [18]). Importantly, decades of research in cognitive neuroscience have yielded considerable insights into the putative neural substrates of error monitoring—linking error processing systems to a medial frontal network comprising the anterior cingulate cortex (ACC), lateral prefrontal cortex (PFC), supplemental motor areas (SMA), and insula (see [11,19] for reviews). Furthermore, this neural network is consistently implicated in the generation of a systematic sequence of event-related potentials (ERPs) after error commission on speeded-choice tasks (e.g., Eriksen flanker tasks). Two of the most reliable and well-studied neural indices of error monitoring are ERPs: the error-related negativity (ERN; [20,21]) and the error positivity (Pe; [15,16]).

The ERN is a frontal central negative deflection that occurs within 100 ms after error commission and has been source localized to the ACC and SMA (see [22] for a review). Although the functional significance of the ERN is still debated, two prominent theories grounded in computational modeling have linked the ERN to early detection of: (1) competing response representations (error vs. correct; i.e., conflict monitoring theory, [14,23]) and; (2) mismatch between predicted and actual performance outcomes (i.e., reinforcement learning theory, [24]). Despite their differences, both theories imply that larger ERN amplitudes are associated with higher acuity in detecting performance-related discrepancies. Additionally, it has been posited that the ERN indexes emotional processing of errors [25,26] on the basis of its association to brain regions implicated in pain and negative affect (e.g., ACC, [27]), psychological disorders characterized by affective dysregulation [28], and affective physiological responses (e.g., skin conductance and startle response, [29,30]). Though the exact role of affect in ERN modulation remains unclear, this line of research raises the possibility that interventions that alter affective processing (e.g., mindfulness meditation) may be liable to modulate the ERN. 

Following the ERN, the Pe is a central parietal positive deflection peaking approximately between 200 and 400 ms post response. Evidence for the localization of the Pe is not as conclusive as the ERN, with studies pointing to the rostral ACC, posterior cingulate, and insula [31,32,33]. Similar to the ERN, three major theories on the functional significance of the Pe have been proposed, including: (1) conscious error recognition [34,35]; (2) responsivity to the motivational significance of the error [21] and; (3) affective processing of conscious errors [36,37]. Although few studies have pitted these theories against each other, accumulating evidence continues to support the Pe as a neural correlate of conscious error awareness whereas comparatively less evidence has been found in favor of the affective processing hypothesis [12,38,39].

In addition to the abundance of basic research on the ERN and Pe, a wide literature base has implicated error monitoring in various regulatory and functional domains such as stress regulation [40,41], impulse control [42,43], attention regulation [44], and academic performance [45,46,47]. Despite the central role of error monitoring in maintaining healthy functioning, surprisingly few studies have examined the intersection between mindfulness and error monitoring. 

In one of the first investigations, Teper and Inzlicht [48] employed a cross-sectional design comparing the ERN, Pe, and behavioral performance between experienced meditators and novice controls. Interestingly, meditators exhibited larger ERN amplitudes and superior accuracy relative to controls. Replicating these findings, Andreu et al. [49] reported enhanced ERN amplitudes and higher accuracy in experienced Vipassana meditators compared to novices. However, a more recent study by Bailey and colleagues [50] utilizing advanced whole-scalp analysis reported no differences in behavioral or ERP indices of error monitoring between experienced meditators and novices. Surprisingly, none of the studies found group differences in Pe amplitude despite the conceptual overlap between the Pe and mindfulness as constructs involving conscious awareness. 

Experimental designs have produced even more divergent outcomes. Using a single-session experimental manipulation, Larson and colleagues [51] found diminished Pe amplitudes but no change in the ERN or behavioral performance after novice non-meditators completed a brief guided mindfulness meditation relative to controls. Contradictorily, a clinical longitudinal study examined the effects of mindfulness-based cognitive therapy (MBCT) on adult ADHD patients, finding that MBCT patients exhibited *increased* Pe amplitudes, but no changes in ERN or behavioral performance [52]. Yet another study compared brief single-session inductions of thought-focused relative to emotion-focused mindfulness practice, reporting increased ERN but no change in Pe in only the emotion-focused group [53]. The relative sparsity of studies combined with the equivocality of the findings signal a need for further clarification into the nature of the mindfulness–error monitoring relationship. 

Toward this end, recent critical reviews of mindfulness research highlight several prescriptive factors that appear prudent to consider [7,9,11,54,55,56,57,58,59,60,61,62,63,64]. First, mindfulness is a polylithic construct that can reflect a dispositional trait, state of mind, mental training modality (e.g., meditation), or psychological intervention. Importantly, such construct heterogeneity challenges standardized operationalizations of mindfulness and may partially explain the different outcomes in the studies reviewed above. For instance, Larson et al. [51] examined mindfulness as a brief guided meditation, whereas Andreu et al. [49] and Teper and Inzlicht’s [48] cross-sectional design operationalized mindfulness as a derivative of meditative experience. Consequently, it is likely that the “acute” effects of mindfulness training in novices differ from the oft-posited “trait-like” changes associated with cumulative meditative experience [15]. Moreover, Schoenberg and colleagues [52] investigated mindfulness in the context of a psychological intervention for ADHD, introducing interpretive complications arising from uncontrolled components of the intervention (e.g., parsing effects of psychoeducation vs. social support vs. mindfulness training) and idiographic factors unique to an ADHD clinical sample. Lastly, Saunders et al. [53] bisected their mindfulness induction to exclusively direct awareness toward either emotions or thoughts, thereby narrowing the scope of inquiry to mindfulness of specific internal states. Such differences in operationalization and sample characteristics (e.g., novice vs. experienced vs. clinical) represent unique methodological challenges extending from construct heterogeneity that, without proper contextualization, can obfuscate understanding of how *different aspects* of mindfulness influence error monitoring. 

Second, there is substantial variation among mindfulness practices. This is perhaps best exemplified by the empirically supported distinction between focused attention (FA) and open monitoring (OM) meditation [65]—two separate meditative practices that are often unwittingly subsumed under the umbrella term “mindfulness meditation”. FA meditation is conceptualized as the voluntary direction of sustained attentional awareness to a target object (e.g., the breath), whereas OM meditation involves non-judgmental monitoring of momentary experience without explicit direction to attend to a preselected target. Importantly, studies comparing FA and OM meditation have shown unique patterns of neural activation [66,67] and different effects on cognitive and affective processes ([68,69]; see [70] for a review). Taken together, evidence supports the possibility that functional differences between OM and FA meditation may extend to the domain of error monitoring.

To date, however, studies of mindfulness and error monitoring have given little consideration for technical variation within mindfulness practice—whether it be in the context of cross-sectional designs involving experienced meditators and novices, brief mindfulness inductions (e.g., one session guided meditation), or multi-week mindfulness training programs. For example, Teper and Inzlicht [48] included participants from a variety of meditative traditions including Vipassana and broadly defined “concentrative traditions”. Vipassana meditation is often considered an OM meditation [66,67,70,71], whereas “concentrative” appears to suggest some form of FA meditation. Similar considerations apply to Schoenberg et al. [52] given that standard protocols for MBCT involve FA- and OM-based practices in addition to experiential exercises that draw from both meditations (see [72] for a systematic dismantling study; [73]). Importantly, such mixing of FA and OM techniques impedes the ability to parse the extent to which distinguishing features of each respective practice relate to error monitoring. For example, Andreu et al. [49] and Larson et al. [51] appeared to homogenize meditative technique, with recruitment of strictly experienced Vipassana meditators in the former, and the employment of a guided breath-oriented FA meditation in the latter. Interestingly, however, Saunders and colleagues’ [53] novel induction seemingly mixed properties of both FA and OM meditation, instructing participants to direct awareness toward a specific *category* of internal experience (thoughts vs. emotions) rather than a fixed target object (as in FA) or any momentary experience (as in OM). Although their study yielded illuminating insights into the specific influence of mindfulness of emotion on error monitoring, the unique nature of the induction challenges whether the conclusions can generalize to FA or OM meditation, two of the most common and standard forms of mindfulness practice. Reviewing these studies through the purview of the FA/OM dichotomy reveals a distinct gap in the literature—namely, that no prospective study has examined the effects of OM meditation on error monitoring.

In addition to supplementing the literature, there are complementary incentives to an experimental investigation of OM meditation, particularly in a novice non-meditating sample. The points reviewed above represent some of the most pressing challenges in mindfulness research—challenges that may be surmounted through active incorporation of the prescriptive recommendations identified by the field (e.g., [7]). Extrapolating this to the relatively unexplored topic of mindfulness and error monitoring, prudent first steps may be to: (1) fill clear gaps in the literature; (2) address extant issues associated with construct heterogeneity, meditation experience, and technical variation; (3) begin development of a standardized, replicable, and generalizable methodology through incremental testing and refinement of measures that are sensitive to various operationalizations of mindfulness. 

Consonant with these steps, the current study sought to examine the effects of a brief guided OM meditation on neural (i.e., ERN, Pe) and behavioral measures of error monitoring in meditation-naïve participants. Measures of trait mindfulness were collected to account for potential group differences in dispositional mindfulness and explore the extent to which individual differences in trait mindfulness relate to error monitoring. Heeding the recommendations of Van Dam and colleagues [7], this approach succinctly circumscribes mindfulness training to a brief guided OM meditation (as opposed to FA or broader training modality involving mixed meditative techniques), minimizes confounds associated with meditative experience, standardizes training duration, and leverages natural variability in trait mindfulness to extend analysis across multiple aspects of mindfulness (i.e., meditative practice and dispositional trait).

Given the mixed findings from the studies reviewed above in addition to the absence of research investigating the effects of OM meditation on error monitoring, we established our predictions using the best available evidence. Regarding the ERN, both Andreu et al. [49] and Teper and Inzlicht’s [48] sample included experienced OM meditators (e.g., Vipassana practitioners) and reported larger ERN amplitudes relative to novices. Furthermore, Saunders and colleagues [53] reported increased ERN amplitudes as a function of directing mindfulness toward emotions relative to thoughts, positing a link between affective awareness and ERN modulation. In this light, that Larson et al. [51] did not observe changes in the ERN may be explained by their employment of a FA as opposed to OM induction. Again, FA meditation involves sustained attentional awareness to a fixed target object and demands redirection of attention away from non-target phenomena—put more directly, breath-oriented FA meditation inherently prioritizes awareness of breath over affective experience. On the other hand, OM meditation emphasizes the fostering of momentary awareness which may include arising emotional states among other forms of internal experience (e.g., physical sensations) [65]. Consequently, it stands to reason that if mindfulness of emotion is central to ERN modulation as suggested by Saunders and colleagues [53]—a unique property of OM relative to breath-oriented FA meditation—then assuming sufficient mindful awareness of emotion is cultivated during practice, a brief OM meditation induction was predicted to increase ERN amplitude. 

With respect to the Pe, the same rationale undergirded our prediction that Pe amplitude would not change—none of the aforementioned studies involving OM meditators [48,49] or unique components of OM meditation [53] reported change in the Pe. Although Larson et al. [51] reported a decrease in the Pe, it seemed unreasonable to expect replication given the previous reflections on the differences between FA and OM meditation, in addition to Schoenberg and colleagues’ [52] inconsistent finding that Pe increased with MBCT training. Lastly, behavioral performance was not expected to differ given the predominance of null findings reported in similar studies employing brief mindfulness inductions on novice samples [51,53,74].

Secondary exploratory analysis examined the relation between trait mindfulness and error monitoring. Although measurement of trait mindfulness remains a topic of considerable debate [55,75,76,77], there appears to be consensus that trait mindfulness contains multiple subfacets. Indeed, the Five Facet Mindfulness Questionnaire [78] is an empirically validated measure that captures five factor-derived facets of trait mindfulness: Observing (FFMQ-O), Describing (FFMQ-D), Acting with Awareness (FFMQ-AA), Nonjudging (FFMQ-NJ), and Nonreactivity (FFMQ-NR). Among these facets, FFMQ-AA measures the propensity to attend to the present moment (e.g., ‘It seems I am “running on automatic” without much awareness of what I’m doing’). Given that on-task attention has been implicated in conceptual models of both the ERN and Pe [14,21], FFMQ-AA exhibits strong theoretical relevance to error monitoring and may be related to the ERN and Pe. This possibility is further supported by previously reported relationships between FFMQ-AA and attention-related ERPs [79,80,81]. Lastly, exploration of FFMQ-NR and FFMQ-NJ seemed to be a natural follow-up on past suggestions implicating nonjudgment in ERN modulation (i.e., increased nonjudgmental awareness of affective error salience; see [48,53]), and nonreactivity in Pe modulation (i.e., reduced error orientation; see [51]), respectively.

## 2. Method

### 2.1. Participants

Two hundred twelve right-handed, native English speaking, female undergraduates participated in the current study as part of a large multi-task experiment aimed at examining the effects of brief OM meditation on emotion regulation and error monitoring. In accordance with the research question, the current study circumscribed the scope of the data to measures of mindfulness and error monitoring. Notably, all participants were novice non-meditators with no mindfulness training experience and did not differ in exposure to the procedures and tasks. We recruited female participants to minimize known confounds related to sex differences in ERPs of emotion processing (i.e., LPP; [82,83]) and error monitoring (i.e., ERN and Pe; [84,85]). Furthermore, emerging studies have reported sex differences in associations involving trait mindfulness [86], responsivity to mindfulness interventions [87,88,89], and the practical likelihood of adopting a meditation practice [90]. 

Prospective participants were screened for neurological disorders and mindfulness training experience—all participants identified as complete novices, endorsing no previous experience. Consented participants were randomized to either a meditation (*n* = 106) or control group *(n* = 106) involving different audio inductions (see below) and remained naïve to group assignments through the end of the experiment. Two participants were excluded from analyses because of failure to follow stimulus–response mapping instructions (see ‘Flanker Task’ section) that produced an error rate exceeding 50%. Four more participants were excluded due to unrecordable (1; hair extensions), unsaved (1; experimenter negligence), and unusable (2; reference channel artifacts resulting in excessive data loss (>80%) after artifact rejection) data. Consequently, the final sample consisted of two hundred and six participants (control: *n* = 103; meditation: *n* = 103), comprising an age range from 18 to 28 (*M* = 19.22, *SD* = 1.34). The majority of the sample identified as Caucasian/White (84.0%), the remaining participants identified as African American/Black (5.3%), Asian (2.4%), Latino/Hispanic (3.9%), Bi-Racial/Multi-Racial (2.9%), or Other (1.5%). No participants discontinued their involvement after consent.

### 2.2. Procedural Overview

The Institutional Review Board at Michigan State University approved the study procedures (IRB #14-871) and all participants provided written informed consent prior to participation. Participants were first fitted with an elastic cap for electroencephalogram (EEG) recording. Continuous EEG was recorded during completion of four sequential tasks: (1) a brief resting task, during which participants were instructed to close their eyes and sit quietly for 5 min; (2) participants were next randomly assigned to complete a guided audio OM meditation or listen to a control audio. To control for potential differences in the proclivity to keep eyes closed or open as a function of experimental condition (i.e., participants in the meditation condition may be more inclined to close eyes), all participants were instructed to keep their eyes closed during the audio induction; (3) immediately after the induction, participants completed an emotion picture viewing task during which they viewed a randomized series of neutral and negative high arousing images; (4) participants next completed a battery of self-report questionnaires and manipulation check measures; (5) lastly, participants completed a computerized flanker task (described below). Participants completed the self-report battery prior to the flanker task because time-sensitive self-report responses were collected to address another research question involving emotion regulation—these data will be reported in a separate future manuscript (Lin et al. in preparation).

### 2.3. Audio Induction

To maintain methodological continuity and cross-study generalizability, the audio inductions were direct replications of material used in previous work [80]. The meditation induction was a 20-min guided OM meditation exercise led by Steve Hickman from the University of San Diego Center for Mindfulness [91]. Consistent with the description of OM meditation presented in the introduction, the recording instructed participants to direct their attention inward, taking notice of present-moment feelings, thoughts, and physical sensations in an open, nonjudgmental manner. 

The control condition involved an 18-min audio recording of a Technology, Entertainment, Design (TED) talk by the linguist Chris Lonsdale [92]. The recording instructed participants how to rapidly develop second language fluency. Importantly, the control audio was selected due to match the didactic style, speech, gender, and duration of the guided meditation. 

### 2.4. Flanker Task

Participants completed an arrow version of the Eriksen Flankers task [93], during which they were seated approximately 60 cm in front of a computer monitor and instructed to respond to the center arrow of 5 arrows that was either congruent (i.e., <<<<< or >>>>>) or incongruent (i.e., <<><< or >><>>) with the surrounding flanking arrows. Characters were presented in standard white font on a black background and subtended 1.3° of the visual angle vertically and 9.2° horizontally. The task was administered on a Pentium R Dual Core computer using the E-Prime program (Psychology Software Tools, Sharpsburg, MD, USA) which presented the stimuli and recorded response measurement.

Arrows were presented for 200 ms during each trial. Participants were given a 950 ms response window before the start of the next intertrial interval. During the intertrial interval, a fixation cross (+) was presented on screen that varied in duration between 600 ms and 1000 ms. The flanker task included 512 total trials separated into 8 blocks of 64 trials. Within each block, half of the trials were congruent and half were incongruent. Left and right mouse buttons corresponded to respective arrow directions, and participants were instructed to respond as quickly and as accurately as possible using either their right index (left button) or middle finger (right button). At the end of each block, performance-based feedback was presented to encourage speed, accuracy, and sufficient error commission [94]. When performance accuracy dropped below 75%, participants were instructed to respond more accurately. If performance exceeded 90%, participants were instructed to respond faster. Accuracy within the 75–90% range prompted the feedback “You’re doing a great job”.

### 2.5. Trait Mindfulness

Trait mindfulness was measured by the 39-item Five Facet Mindfulness Questionnaire (FFMQ; [78]), a psychometrically validated scale that differentiates dispositional mindfulness into five facets. The five facets include: (a) observing (FFMQ-O), defined as noticing inner and external experiences; (b) describing (FFMQ-D), defined as verbal articulation of inner experiences; (c) acting with awareness (FFMQ-AA), defined as attending to present moment experience; (d) nonjudging (FFMQ-NJ), defined as adopting a nonevaluative attitude toward internal experiences, and (e) nonreactivity (FFMQ-NR), defined as permitting experiences to occur and go without attachment or elaboration. Participants responded to the 39 items using a 5-point Likert scale ranging from 1 (*never or very rarely true*) to 5 (*very often or always true*). 

### 2.6. Manipulation Check

A post-session manipulation check questionnaire from Lin et al. [80] was used to assess for potential differences in engagement and receptivity to the experimental manipulation. Participants reported the degree to which the audio induction was engaging, interesting, and arousing (1 = *not at all*, 7 = *very*). Participants also indicated their comprehension level (1 = *did not understand*, 7 = *completely understand*), emotional reaction (1 = *very negative*, 4 = *neutral*, 7 = *very positive*), and extent of learning (1 = *very little*, 7 = *very much*). Lastly, because novice meditators may be particularly susceptible to sleepiness and drowsiness during meditation [95], participants were asked to report their sleepiness (1 = *feeling active, vital, alert, or wide awake*, 8 = *I fell asleep*) using the Stanford Sleepiness Scale [96].

### 2.7. Psychophysiological Recording and Data Reduction

Continuous electroencephalographic activity was recorded from a 64-channel stretch lycra cap using the BioSemi ActiveTwo system (BioSemi, Amsterdam, The Netherlands). Recordings were derived from 64 Ag-AgCl electrodes arranged in the 10/20 system. An additional two electrodes were placed on the left and right mastoids to serve as reference. Blinks and eye movements generating electrooculogram (EOG) activity were recorded at FP1 and at three electrodes placed under the left pupil and to the left and right outer canthi. Per BioSemi’s design specifications, the Common Mode Sense (CMS) active electrode and Driven Right Leg (DRL) passive electrode formed the ground during data acquisition. All signals were digitized at 1024 Hz. 

**Figure 1:**
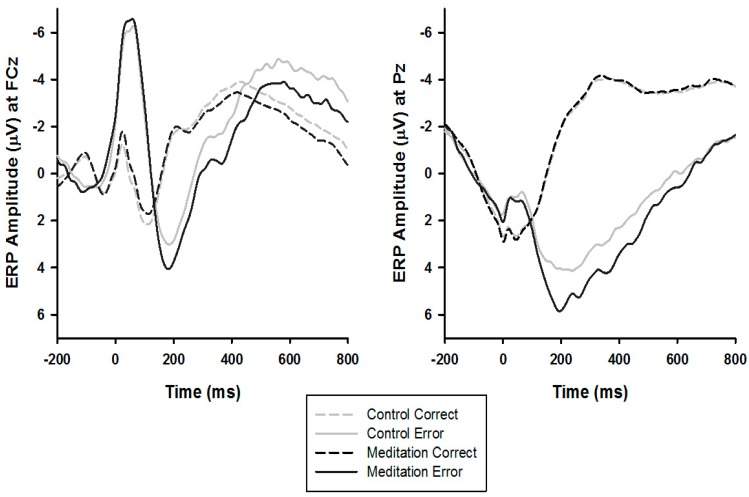
Grand average waveforms as a function of group representing the error-related negativity (ERN; left) averaged across frontocentral electrode site FCz and the error positivity (Pe; right) averaged across central electrode site Pz.

**Figure 2:**
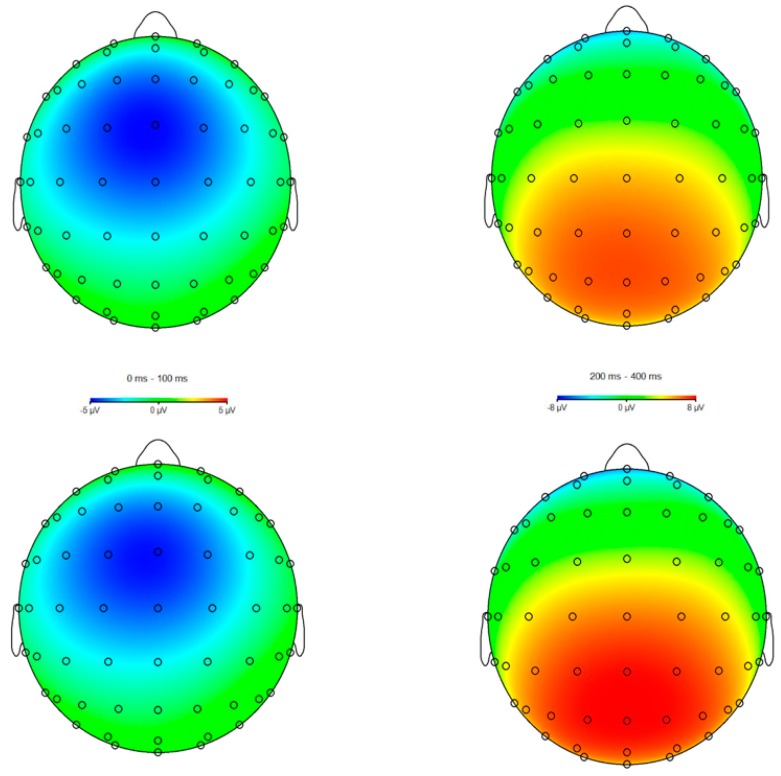
Scalp voltage maps for the error-minus-correct error-related negativity (ERN, left) and error positivity (Pe, right) as a function of group (control, top; meditation, bottom).

BrainVision Analyzer 2 (BrainProducts, Gilching, Germany) was used to conduct offline analyses. Scalp recordings were referenced to the mean of the mastoids and band-pass filtered with cutoffs of 0.1 and 30 Hz (12 dB/oct rolloff). The regression method developed by Gratton, Coles, and Donchin was used to correct for Ocular artifacts [97]. Consistent with established guidelines and past work from our laboratory [80,81,98,99,100,101], physiological artifacts were removed using an algorithm such that trials that met the following criteria were rejected: a voltage step exceeding 50 μV between contiguous sampling points, a voltage difference greater than 300 μV within 200 ms intervals, voltage exceeding ± 200 μV, or a maximal voltage difference less than 0.5 μV within 100 ms intervals. Also consistent with previous work [80,81,98], ERPs were locked to response onset, with a 200 ms pre-trial baseline correction. Response-locked data were segmented into individual epochs beginning 200 ms before response onset and continuing for 800 ms following the response. In line with the collapsed localizer method [102] and standard time window estimates [21,22], the ERN and Pe were then quantified as the difference in average activity between error and correct trials occurring between 0–100 ms and 200–400 ms post-response onset at recording sites FCz and Pz, respectively—where the amplitude was statistically determined to be maximal (see Figure 1 and Figure 2). All data reduction parameters (e.g., electrode, baseline, and time window selection) were determined prior to data analysis and never modified for re-analysis or exploratory means.

### 2.8. Data Analyses Overview

Self-report, behavioral, and ERP statistical analyses were conducted using IBM SPSS Statistics (Version 23.0). To ensure no group differences in baseline trait mindfulness and compliance to the experimental procedures, FFMQ and manipulation check responses were submitted to independent samples *t*-tests with Group (meditation vs. control) as the between-subjects variable. 

Behavioral data were submitted to paired t-tests and repeated measures analysis of variance (rANOVAs). Effect size estimates in ANOVA models were reported using partial eta squared $\eta_{p}^{2}$ where 0.01 represents a small effect, 0.06 a medium effect, and 0.14 a large effect [103,104]. Number of errors were submitted to a paired *t*-test comparing performance accuracy as a function of Congruency (congruent vs. incongruent). Independent samples *t*-tests were then conducted to determine whether groups differed in the number of overall errors and errors by trial congruency. RTs were submitted to a 2 (Response Type: error vs. correct) × 2 (Congruency: congruent vs. incongruent) rANOVA with Group (meditation vs. control) as a between-subjects factor. Paired samples *t*-tests were conducted to aid interpretation when significant interactions emerged. Degrees of freedom varied among the *F*-tests because of performance variability (e.g., participants committing no errors on congruent trials are excluded from analyses involving congruency). To examine post-error performance, RTs and accuracy were submitted to two separate one factor (Response Type: post-error vs. post-correct) rANOVAs with Group (meditation vs. control) as a between-subjects factor. 

For ERP analyses, ERN and Pe amplitude were likewise submitted to two separate one factor (Response Type: error vs. correct) rANOVAs with Group (meditation vs. control) as a between-subjects factor. Lastly, bivariate correlations separated by group were conducted to explore relationships between trait mindfulness, behavioral performance, and ERPs. Follow-up Fisher r-to-z tests of independent correlations were conducted to determine if significant relationships differed by group. 

## 3. Results

### 3.1. Baseline Mindfulness and Manipulation Check

Descriptive statistics of all measures by group are presented in Table 1. As expected, there were no group differences in any facet of trait mindfulness, or in overall mindfulness (*t*s < |1.49|, *p*s > 0.14).

Participant responses on the manipulation check revealed group differences in interest (*t*(1, 204) = 4.32, *p* < 0.01), learning (*t*(1, 204) = 6.02, *p* < 0.01), and sleepiness (*t*(1, 204) = −2.76, *p* < 0.01), such that relative to the meditation group, participants in the control group rated the control audio as more interesting (control: *M* = 4.54, *SD* = 1.62, meditation: *M* = 3.55, *SD* = 1.67), indicated learning more (control: *M* = 4.68, *SD* = 1.36, meditation: *M* = 3.51, *SD* = 1.42), and endorsed less sleepiness (control: *M* = 3.81, *SD* = 1.40, meditation: *M* = 4.36, *SD* = 1.48). Importantly, there were no differences in engagement, arousal, emotional reactivity, or understanding (*t*s < |1.92|, *p*s > 0.06), suggesting that although groups differed in their experiential appraisal of the audio inductions, participants nonetheless approached the task with equal levels of engagement and comprehension. Notably, with the exception of sleepiness which was not previously measured, this constellation of group differences fully replicated Lin et al. [80]. To determine whether the unexpected group difference in self-reported interest, learning, and sleepiness confounded the results of the study, all analyses were re-run with the three variables entered as continuous covariates. All output remained the same with respect to statistical significance and effect size. Therefore, the results are henceforth presented in accordance to what was originally described in the methods.

### 3.2. Behavioral Data

Descriptive statistics for behavioral and ERP data are presented in Table 2. Overall flanker task accuracy was relatively high (*M* percent correct = 82.87%, *SD* = 8.73%). Participants made an average of 80.15 errors (*SD* = 41.38), with more errors on incongruent trials (*M* = 56.26, *SD* = 29.97) than congruent trials (*M* = 23.89, *SD* = 19.18, *t*(205) = 16.22, *p* < 0.01). Importantly, there were no group differences in overall errors or errors by trial congruency (*t*s < 0.90, *p*s > 0.37).

The analysis of RTs revealed main effects of Response Type and Congruency, such that RTs on error trials (*M* = 331.15, *SD* = 48.22) and congruent trials (*M* = 379.47, *SD* = 44.56) were faster than on correct (*M* = 410.55, *SD* = 46.52, *F*(1, 203) = 1236.88, *p* < 0.01, $\eta_{p}^{2}$ = 0.86) and incongruent trials (*M* = 418.95, *SD* = 52.16, *F*(1, 203) = 529.00 *p* < 0.01, $\eta_{p}^{2}$ = 0.72), respectively—consistent with known speed-response type and speed-congruency trade-offs. These main effects were qualified by a significant Response Type X Congruency interaction (*F*(1, 203) = 107.39, *p* < 0.01, $\eta_{p}^{2}$ = 0.35), such that RT differences between incongruent and congruent trials were larger on correct trials (*M* = 54.29, *SD* = 25.12) relative to error trials (*M* = 23.24, *SD* = 38.23, *t*(204) = 10.31, *p* < 0.01). Notably, there were no significant interactions involving Group (*F*s < 2.97, *p*s > 0.09), indicating that there were no group differences in RTs. 

In keeping with the typical post-error slowing (PES) effect, analyses revealed faster RTs following correct responses (*M* = 394.85, *SD* = 47.06) than following errors (*M* = 421.04, *SD* = 62.16, *F*(1, 204) = 134.99, *p* < 0.01, $\eta_{p}^{2}$ = 0.40). Critically, there was no Response Type X Group interaction (*F*(1, 204) < 0.01, *p* = 0.99, $\eta_{p}^{2}$ < 0.01), indicating that PES did not differ by group. The analysis of post-error accuracy (PEA) revealed a main effect of Response Type, such that accuracy following correct responses (*M =* 84.54%*, SD =* 7.07) was slightly higher than accuracy following errors (*M* = 82.82%, *SD* = 13.75, *F*(1, 204) = 4.80, *p* = 0.03, $\eta_{p}^{2}$ = 0.02). Again, there was no Response Type X Group interaction (*F*(1, 204) = 0.96, *p* = 0.33, $\eta_{p}^{2}$ < 0.01), suggesting no group differences in PEA.

### 3.3. ERPs

The analyses involving ERN amplitude revealed an expected main effect of Response Type (*F*(1, 204) = 416.50, *p* < 0.01, $\eta_{p}^{2}$ = 0.67), reflecting larger negativity on error trials (*M* = –5.16, *SD* = 3.59) relative to correct trials (*M* = 0.01, *SD* = 2.72). There was, however, no significant Response Type X Group interaction (*F*(1, 204) = 0.06, *p* = 0.81, $\eta_{p}^{2}$ < 0.01), indicating that ERN amplitude did not differ by group. 

Similarly, the main effect of Response Type on Pe amplitude was significant (*F*(1, 204) = 574.74, *p* < 0.01, $\eta_{p}^{2}$ = 0.74), revealing increased positivity on error trials (*M* = 4.00, *SD* = 4.14) relative to correct trials (M = –3.43, SD = 2.85). Critically, there was a significant Response Type X Group interaction (*F*(1, 204) = 4.62, *p* = 0.03, $\eta_{p}^{2}$ = 0.02), such that the Pe was larger in the meditation group (*M* = 8.10, *SD* = 4.19) relative to controls (*M* = 6.77, *SD* = 4.70; *t*(204) = 2.15, *p* = 0.03). For full transparency, the magnitude of this interaction was reduced after re-running the model with interest, learning, and sleepiness as continuous covariates (*F*(1, 204) = 4.50, *p* = 0.04, $\eta_{p}^{2}$ = 0.02). However, the effect size and associated interpretive significance remained unchanged.

### 3.4. Relationships between ERPs, Behavioral Performance, and Trait Mindfulness

Given that the meditation group exhibited larger Pe amplitude, relationships between ERPs and behavioral performance measures were examined across groups. Correlations among the ERN, Pe, error rate, error RT, correct RT, PES, and PEA separated by group are presented in Table 3. For both groups, larger (more negative) ERN amplitudes were associated with fewer errors (controls: *r* = 0.31, *p* < 0.01; meditation: *r* = 0.26, *p* < 0.01), faster RTs on error (controls: *r* = 0.33, *p* < 0.01; meditation: *r* = 0.30, *p* < 0.01) and correct trials (controls: *r* = 0.23, *p* = 0.02; meditation: *r* = 0.31, *p* < 0.01), greater PEA (controls: *r* = −0.36, *p* < 0.01; meditation: *r* = −0.31, *p* < 0.01), but was unrelated to PES (controls: *r* = 0.03, *p* = 0.75; meditation: *r* = −0.02, *p* = 0.86). Similarly, larger Pe amplitudes were associated with fewer errors (controls: *r* = −0.42, *p* < 0.01; meditation: *r* = −0.37, *p* < 0.01), faster error RT (controls: *r* = −0.26, *p* < 0.01; meditation: *r* = −0.29, *p* < 0.01), greater PEA (controls: *r* = 0.41, *p* < 0.01; meditation: *r* = 0.40, *p* < 0.01), but were unrelated to correct RT or PES (controls: *rs* < 0.12, *ps* > 0.24; meditation: *rs* < |0.1|, *ps* > 0.33). Notably, all listed correlations between ERPs and behavioral measures did not differ by group (*z*s < |0.61|, *p*s > 0.52).

In keeping with the secondary exploratory analysis, ERPs were examined in relation to the five facets of trait mindfulness as a function of group. Relationships among the Pe, ERN, and FFMQ are presented in Table 4. Surprisingly, across both groups, none of the FFMQ subfacets related to the ERN (controls: rs < |0.05|, ps > 0.60; meditation: rs < 0.15, ps > 0.13) or Pe (controls: rs < |0.11|, ps > 0.26; meditation: rs < 0.14, ps > 0.16). 

## 4. Discussion

Previous studies have yielded unique findings toward understanding the relationship between mindfulness and error monitoring. Taken as a whole, the mixed and often contrasting nature of the available evidence obfuscates the drawing of clear and generalizable conclusions. A careful review of the nascent literature underscored the methodological and interpretive challenges associated with mindfulness research (e.g., construct heterogeneity) and revealed clear gaps in knowledge (e.g., how OM, as opposed to FA, meditation affects error monitoring). In the largest sample to date (to our knowledge), the present study sought to address these issues by examining the effects of a brief guided OM meditation on neural (i.e., ERN and Pe) and behavioral indices of error monitoring. Importantly, we restricted the sample to novice non-meditating participants and measured trait mindfulness in order to minimize known confounds associated with meditative experience and account for individual variation in dispositional mindfulness, respectively. Notably, our findings did not support our hypotheses—contrary to our prediction that the meditation group would exhibit a larger ERN and no change in the Pe, the ERN did not differ between groups but the Pe was larger in the meditation group.

More broadly, our findings showed that a single session of guided OM meditation modulates ERP but not behavioral measures of error monitoring. Again, participants who completed the OM meditation exhibited no change in the ERN, but a small effect was observed in Pe amplitude, such that meditators showed a larger Pe relative to controls. Analysis of behavioral performance including error rates, RTs, PEA, and PES revealed no group differences. Consistent with the common, but debated, suggestion that larger ERN and Pe amplitudes reflect better cognitive ability (see [105] for a brief review), we found that larger ERN and Pe amplitudes were indeed associated with fewer errors, faster RTs, and greater PEA across both groups. Lastly, exploratory analysis revealed no relationships between the subfacets of trait mindfulness and the ERN or Pe. 

### 4.1. ERN Modulation

Contrary to expectations, the OM meditation did not increase the ERN. Previous studies using similar single-session meditation inductions yielded equivocal findings, with Larson and colleagues [51] reporting no modulation of the ERN, whereas Saunders et al. [53] found increased ERN amplitudes. Given functional theories implicating the ERN in affective processing [25,26] in addition to Saunders and colleague’s [53] suggestion that mindfulness of emotion is central to enhancing the ERN, we reasoned that a guided OM meditation containing instructions to attend to arising emotional states (as opposed to the FA meditation employed by [51]), may likewise increase the ERN. In light of the null finding, however, this line of reasoning is in clear need of reexamination. Toward this end, one potentially important point of consideration involves procedural and technical differences among the inductions.

More precisely, the mindfulness inductions employed in the present study and in Larson et al. [51], though comparatively unique with respect to their OM and FA properties, followed the format of a prototypic guided meditation. In contrast, the experimental manipulation in Saunders et al., [53] begins similarly with a guided “concentrative meditation practice” (p. 97) but diverges to instruct insular awareness on either emotions or thoughts, and concludes with a post-induction writing exercise to reinforce an emotion or thought focused mental state. Notably, the latter parts of their induction represent nontrivial deviations from traditional forms of guided FA or OM meditation insofar that the exclusive emphasis on promoting emotional awareness may have played a key role in augmenting the ERN. 

Extrapolating this suggestion to the current study, the relatively indiscriminate nature of OM meditation may not facilitate sufficient attentional insularity towards emotion to modulate the ERN in the manner suggested by Saunders and colleagues [53]. Indeed, OM meditation involves directing attention to *any* present moment phenomena. Thus, novices following the technical instruction of the present study likely adopted a broader mode of attentional awareness that encompassed different aspects of internal experience (e.g., breath, posture, or physical sensation) *in addition* to emotions. Given these considerations, whether long-term iterative practice of OM meditation would enhance the ERN as suggested by the experienced meditators in Andreu et al. [49] and Teper and Inzlicht [48], remains an open and intriguing empirical question. In addressing this question, it may be imperative to directly test the proposition that ERN modulation is contingent on the degree of emotional awareness cultivated during mindfulness training. Nonetheless, the current findings demonstrate that a single session of OM meditation does not modulate the ERN in novice non-meditators. 

### 4.2. Pe Modulation

A second unexpected finding was that OM meditation participants exhibited larger Pe amplitudes relative to controls. This contrasts the findings of Saunders et al. [53] and Larson et al. [51], with the former reporting no modulation and the latter observing *diminished* Pe amplitudes. Here again, subtle but potentially significant differences in technical instruction—namely, variation in scope and object of awareness during mindfulness training—may underlie the mixed findings. 

Specifically, Larson et al. [51] and Saunders’ et al. [53] inductions emphasized directing attention toward a fixed target (breath) or discrete category of experience (emotions or thoughts), thereby promoting a narrow scope and selective object of attentional awareness. OM meditation, on the other hand, fosters the development of a wide and relatively undirected mode of awareness toward the full spectrum of arising experience [65,70]. From this perspective, there appears to be greater conceptual overlap between OM meditation and the purported functional significance of the Pe—namely, the facilitation of conscious awareness toward unfolding momentary events (e.g., an error), as opposed to the sustainment of attention toward a predetermined object found in FA meditation (e.g., the breath). Importantly, the practice of OM meditation may preferentially recruit neural processes involved in conscious error detection, and it may be this selective training of overlapping neural substrates during OM meditation that is evidenced by a larger Pe on the subsequent flanker task. 

Interestingly, theoretical reviews drawing from a wide range of empirical studies have linked Pe generation to the anterior insula [33,106], suggesting that the Pe may reflect the degree of interoceptive awareness to autonomic changes accompanying an error. Of critical relevance, fMRI studies have consistently identified increased activation in the insula during OM meditation but not FA meditation (see [66] for a meta-analysis), supporting the prevailing notion that OM meditation fosters interoceptive awareness. Taken together, it stands to reason that the training of present moment somatosensory awareness during OM meditation may transfer “off-the-cushion” to enhance interoceptive awareness of errors on the flanker task.

On the other hand, FA meditation is reliably associated with activations in cognitive control regions that is thought to underlie voluntary regulation of sustained attention [66]. Interestingly, sustained attention to regulated respiratory processes like the breath is known to engender relaxation and reductions in autonomic arousal (see [107,108]). Remarkably, this effect appeared evident in Larson and colleagues’ [51] study, such that FA meditation produced marked reductions in blood pressure that persisted past completion of the flanker task. In this light, the reduced Pe reported by Larson et al. [51] may reflect a unique effect of FA meditation, insofar that sustained attention on the breath increased global parasympathetic activity across the experimental session. Consequently, the demand to focus attention on the breath in conjunction with broadband attenuation of the autonomic system may have restricted both the scope of internal awareness and the amount of somatic arousal during flanker task performance—collectively *reducing* the degree of error awareness. 

An intriguing caveat to this discussion, as Ullsperger and colleagues [33] thoughtfully expounded, is whether the Pe functionally reflects the *generation* of error-related arousal, or the *degree of awareness* of said arousal. Therefore, to the extent that OM meditation does indeed modulate the Pe through enhancing interoception of errors, it remains unknown whether this effect is maintained by increased generation or awareness of arousal (or both). It is plausible that relationships between autonomic measures and the Pe may vary with training duration and meditative experience. Relevantly, a recent study reported that both FA and OM meditation produced enhanced parasympathetic activation (i.e., less arousal) in long-term monastic practitioners [109], providing some tangential evidence that the increased Pe observed in the current study may functionally reflect increased awareness, rather than enhanced generation, of error-related arousal. 

### 4.3. Trait Mindfulness and the ERN/Pe

None of the subfacets of trait mindfulness related to the ERN or Pe, engendering consideration for why, despite conceptual overlap (e.g., attentional awareness linking FFMQ-AA with Pe), no relationships emerged. Given that the current findings are derived from one of the largest sample sizes to date, one of the most parsimonious interpretations is that trait mindfulness is simply unrelated to the ERN/Pe in novice non-meditators. Following this suggestion, the ERN and Pe might index different neurocognitive processes than what is measured by the FFMQ. It is, however, implausible that the construct of trait mindfulness and the ERN/Pe are entirely unrelated given the range of studies reporting modulation of the ERN/Pe as a function of mindfulness training or meditative experience [48,49,51,52,53]. With that said, measurement of self-reported mindfulness in novice non-meditating samples has been a major point of debate in the contemplative science literature [7,55,62,75,110,111,112]. Consequently, we draw from these sources to offer a possible explanation—that employing self-report measures of mindfulness in a novice sample may carry unique caveats and limitations that complicate thorough assessment of the mindfulness error monitoring relationship. 

Specifically, it has been argued that novices are prone to misidentification and overestimation of personality characteristics relating to mindfulness and attention [76]. For example, non-meditators may construe ruminative self-focus as mindfulness, when such behavior is better accounted for by another construct such as neuroticism. Consistent with this notion, unexpected positive correlations between FFMQ-O, dissociation, psychological symptoms, and thought suppression have been observed in novice samples [78]. Furthermore, novice and meditator response patterns have been shown to differ such that novices are comparatively less likely to endorse negatively, as opposed to positively, worded items (e.g., FFMQ-13 “I am easily distracted”, [78]), suggesting that novices may interpret negatively worded items as indicative of attentional deficits rather than variance in mindfulness [77]. In sum, while trait mindfulness measures yield adequate psychometric properties [113], some evidence suggests that novices may not interpret and respond to certain scale items in ways that reflect the latent construct of mindfulness.

Critically, this difference in item response pattern might alter expected relationships between the subfacets of trait mindfulness and theoretically related neural indices (e.g., FFMQ-AA and Pe). Given the robust relationships observed between ERP and behavioral performance measures here, it is possible that the ERN and Pe may be better estimates of a novice’s “actual” cognitive ability relative to their self-appraisal on the FFMQ. Furthermore, it remains unknown whether engagement in the audio induction and emotional picture viewing task prior to completing the FFMQ altered response patterns. Evidence from Goldberg and colleagues [75] lends credence to this possibility, showing that participants tend to report higher scores on the FFMQ after completing an active intervention—irrespective of whether they received any actual mindfulness training. Importantly, these findings underscore methodological issues that lie beyond the scope of what the present study was designed to address—namely, that self-report measures of mindfulness have been shown to inconsistently differ or change in populations and contexts unrelated to mindfulness and thereby prompt concerns over their validity. Further research is necessary to assess the conditional validity of self-report mindfulness scales by comparing across groups (e.g., novice vs. expert meditators, clinical vs. non-clinical samples) and measuring the extent of convergence with multimodal measures (e.g., behavioral, neural) of theoretically related constructs. 

### 4.4. Methodological Implications, Limitations, and Future Directions 

In reviewing the current findings in relation to the literature, an emergent key point is that subtle technical differences in mindfulness training may play a significant role in producing disparate outcomes in studies of error monitoring. Strikingly, of all the experimental studies investigating mindfulness training in novice samples to date (including the present study), no two findings have been the same with regards to reported change in the ERN and Pe. Critically, a careful examination of the mindfulness inductions employed in this nascent line of research suggests that variation in the scope and object of awareness during mindfulness training may differentially modulate error monitoring. The influence of such technical variation is evident in the extant distinction between FA and OM meditation, wherein the development of a narrow scope of awareness to a singular predetermined target object (e.g., the breath) characteristic of FA meditation has been shown to reduce the Pe [51], whereas the cultivation of a wide scope of awareness to a range of unfolding momentary phenomena during OM meditation was found here to *increase* the Pe. 

Furthermore, the degree to which technical variation influences outcome measures may extend beyond the traditional FA vs. OM dichotomy. For example, modifying the scope and object of awareness from the breath to a broad category of internal experience (e.g., emotions vs. thoughts) as in Saunders et al. [53] appears to produce unique effects on the ERN but not the Pe. Moreover, although most mindfulness training studies have anchored the object of awareness to internal states or processes, other traditional forms of concentrative meditation (e.g., trataka, see [114]) involve directing awareness toward external objects such as a painted dot or candle flame. Such distinction between internal and external objects of awareness may be indicative of yet another technical dichotomy worth further investigation. Taken together, our findings support the developing idea that FA and OM meditation each involve unique neural and functional properties [65,70], and more broadly, demonstrate that accounting for variation in scope and object of awareness during mindfulness training may be critical towards understanding its effects.

Future studies are broadly encouraged to expand upon the suggestions advanced above. One immediate direction is to test the posited differences between OM and FA meditation on error monitoring by comparing them directly. In considering such work, it may be important to note the limitations of the current study. The first and most obvious limitation is that the study was comprised of an all-female sample. Because recent studies have suggested possible sex differences in responsivity to mindfulness training [87,88,89], it appears prudent for future research to determine the extent to which the current findings and associated postulations generalize to males. Second, future designs may consider employing probe measures of state mindfulness (specifically assessing for the scope and object of mindful awareness) in between blocks of the flanker task (see [115] for a similar procedure involving state worry) to elucidate the extent to which detected changes in error monitoring are attributable to induced levels of state mindfulness after meditation and into task performance. Such studies may further parse construct heterogeneity, shedding light on how mindfulness as a meditative practice, dispositional trait, and state of mind interact to influence dependent measures of interest. Third, future research may also benefit from including autonomic measures (e.g., blood pressure, skin conductance, heart rate) that span across mindfulness training and into performative tasks, providing a potential window into how different mindfulness training modalities may distinctively modulate arousal to affect subsequent task performance and error monitoring.

Fourth, it cannot be understated that a single session of guided meditation represents arguably the shortest possible training duration and is inherently limited in producing meaningful inferences about mindfulness training. Indeed, the administration of our induction to a novice non-meditating sample may not have met the implicit assumption that participants were correctly practicing OM meditation. This may engender skepticism over whether the reported Pe modulation pertains to the actual practice of meditation or reflects the effect of a confounding mechanism (e.g., altered task engagement or motivational approach as a function of priming effects on personality characteristics, although controlling for differences between groups in sleepiness, interest, and learning did not alter the results). Relatedly, there is limited evidence to suggest that our current findings and associated postulations are generalizable to prolonged mindfulness training. Consequently, it is unknown whether or how iterative practice of FA or OM meditation would affect neural and behavioral indices of error monitoring, and we strongly caution against the interpretation of our findings as equivalent to effects of extended mindfulness practice. If anything, cross-sectional studies show that experienced meditators do not exhibit demonstrable differences in Pe amplitude [48,49,50]. With that said, we view our work as the beginning of a systematic effort to develop the foundations needed to generate empirically grounded hypotheses for future studies. As mentioned above, directly comparing FA and OM meditation and their respective effects on the Pe appears to be the natural extension to this work. As part of this follow up, because past research has suggested that the Pe can be separated into functionally distinct early and late time [116,117,118,119], exploratory examination of this distinction in relation to mindfulness may be worthwhile. Future longitudinal investigations can examine the extent to which the Pe is modulated over extended training of FA and OM meditation, and how such changes might relate to behavioral performance. 

Last but certainly not least, our findings are limited by the scope of our methodology and analytic approach. Most notably, the unexpected nature of our results (i.e., inconsistency with our a priori hypotheses) in conjunction with the small effect size of the reported Pe modulation and lack of behavioral performance discrepancies across groups challenge the strength of our findings and cast doubt over the postulations advanced above. Such skepticism is compounded by our EEG methodology, which used single-electrode analysis and selective estimation of filtering parameters, baseline correction periods, and ERP time windows. Moreover, it is worth noting that nearly all of the studies reviewed here utilized a similar approach, and therefore variation in EEG analytic parameters may equally contribute to variability in findings (see [50] for an incisive critique). Consequently, it goes without saying that there is a need for replication and explicit testing of the proposed follow-up studies. Specifically, future studies are encouraged to leverage more rigorous EEG processing and analysis methodology to remediate the analytic limitations contained in this study in order to further buffer against the possibility of obtaining spurious findings (see [120] for guidelines). Toward this end, Bailey et al. [50] offers an exemplary demonstration, applying cutting-edge EEG analytic tools which leverage whole-scalp analysis while controlling for multiple comparisons across varying time windows (see [121,122] for open source software). 

Ultimately, future studies designed with consideration of issues in both EEG methodology and meditative practice appear highly fruitful and could lead to a more thorough and definitive understanding of the relationship between mindfulness and error monitoring. Relatedly, it may also be fruitful to examine the extent to which relationships between trait mindfulness and error monitoring are contingent on meditative experience or training duration. Indeed, multiple critiques have cautioned that similar or even identical measures may reflect different latent constructs and or underlying neural processes depending on the expertise of the sample [7,54,76,123,124]. Moreover, it seems plausible that as the effects of iterative practice develop, including presumed changes in trait mindfulness, the resultant relationship between trait mindfulness and error monitoring would likewise change over time. A valuable first step toward testing these suggestions may be to determine whether relationships between trait mindfulness and ERP measures of error monitoring are dissociable between advanced practitioners and novice non-meditators. Later studies could utilize longitudinal approaches to delineate the extent to which such relationships change along the continuum of training experience.

## 5. Conclusions

In sum, our study is a response to the growing calls to carefully parse apart mindfulness, with a specific eye toward explicit denotation and operationalization of the technicalities involved in the broad umbrella term “mindfulness training” (e.g., scope and object of awareness, training duration, sample experience). Critically, the aforementioned considerations extend beyond the study of error monitoring. As we have posited above, technical differences during mindfulness training may involve and affect a range of neurobiological systems. This suggestion dovetails with an evolving and increasingly nuanced literature, in which the neurobiological correlates and putative mechanisms of mindfulness training appear contingent on a host of conceptual, methodological, technical and sample dependent factors—a perspective aptly illustrated by the panoply of divergent findings reviewed here and in the literature more broadly. From this vantage point, future efforts aimed at developing a mechanistic understanding of mindfulness may be well served to not only clearly define what mindfulness is but also carefully consider the technical factors that comprise its training. 

## Figures and Tables

**Table 1:** Means and standard deviations of self-report battery by group.

Variable	Control *N* = 103			Meditation *N* = 103		
	*Range*	*M*	*SD*	*Range*	*M*	*SD*
FFMQ Overall	2.23–4.49	3.21	0.43	2.21–4.23	3.19	0.43
FFMQ-O	11–37	25.45	5.14	17–40	26.52	5.22
FFMQ-D	9–36	26.21	5.39	12–38	26.14	5.68
FFMQ- AA	14–39	27.56	5.72	12–39	26.49	5.74
FFMQ-NJ	9–38	26	6.91	10–38	25.42	6.50
FFMQ-NR	11–34	19.93	4.50	12–30	19.67	3.92
Audio Engagement	1–7	4.23	1.41	1–7	4.17	1.60
Audio Interest	1–7	4.54	1.62	1–7	3.55	1.67
Audio Emotion Reactivity	2–7	4.76	0.99	1–7	4.49	1.07
Audio Arousal	1–6	3.14	1.53	1–6	2.79	1.52
Audio Understanding	1–7	5.43	1.36	1–7	5.43	1.60
Audio Learning	1–7	4.68	1.36	1–6	3.51	1.42
Audio Sleepiness	1–6	3.81	1.40	1–7	4.36	1.48

**Note:** FFMQ, Five Factor Mindfulness Questionnaire (high scores indicate higher levels of dispositional mindfulness, overall score computed as average of all items); FFMQ-O, Observe; FFMQ-D, Describe; FFMQ-AA, Acting with Awareness; FFMQ-NJ, Nonjudgment; FFMQ-NR, Nonreactivity.

**Table 2:** Summary of behavioral and event-related potential (ERP) measures.

Variable	Meditation *N* = 103			Control *N* = 103		
	*SD*	*M*	*Range*	*SD*	*M*	*Range*
Accuracy	0.08	0.83	0.52–0.96	0.1	0.82	0.51–0.96
Number of errors	36.04	77.92	17.235	46.18	82.38	18–243
Incongruent errors	23.07	54.37	11–134	35.58	58.15	13–240
Congruent errors	18.48	23.55	0–105	19.94	24.23	1–107
Error RT (ms)	42.21	327.57	264.87–498.57	53.53	334.73	253.55–520.65
Correct RT (ms)	41.25	408.45	309.82–540.72	51.36	412.65	316.21–568.46
Incongruent error RT (ms)	42.74	332.68	266.12–509	53.73	340.78	257.69–534.46
Incongruent correct RT (ms)	45.38	438.87	322.27–601.92	54.95	441.3	332.88–598.06
Congruent error RT (ms)	50.91	313.44	249.08–511.41	59.98	313.85	233.88–574.64
Congruent correct RT (ms)	39.09	382.89	299.50–496.23	50.39	388.45	296.61–543.34
PES (ms)	33.87	26.22	−210.7	30.76	26.16	−180.66
PEA (ms)	0.13	0.84	0.23–1	0.15	0.82	0.24–1
CRN amplitude (µV)	2.44	−0.23	−14.73	2.97	0.25	−23.96
ERN amplitude (µV)	3.51	−5.34	−19.04	3.67	−4.98	−21.32
Δ ERN (µV)	3.53	−5.11	−18.22	3.74	−5.23	−17.1
Ce amplitude (µV)	2.95	−3.48	−14.42	2.76	−3.39	−14.33
Pe amplitude (µV)	4.11	4.63	−25.37	4.1	3.38	−23.6
Δ Pe (µV)	4.19	8.1	−28.91	4.7	6.77	−25.47

**Note:** RT, reaction time; PES, post-error slowing; PEA, post-error accuracy; CRN, correct-related negativity; ERN, error-related negativity; Pe, post-error positivity; difference, error minus correct.

**Table 3:** Bivariate correlations among ERP measures and behavioral performance by group.

**Control**	**1.**	**2.**	**3.**	**4.**	**5.**	**6.**	**7.**
1. Δ ERN	--						
2. Δ Pe	−0.19	--					
3. Number of errors	0.31 **	−0.42 **	--				
4. Error RT	0.33 **	−0.26 **	0.01	--			
5. Correct RT	0.23 *	−0.08	−0.41 **	0.71 **	--		
6. PES	0.03	0.12	−0.39 **	0.31 **	0.31 **	--	
7. PEA	−0.36 **	0.41 **	−0.86 **	−0.19	0.23 *	0.36 **	--
**Meditation**	**1.**	**2.**	**3.**	**4.**	**5.**	**6.**	**7.**
1. Δ ERN	--						
2. Δ Pe	−0.20 *	--					
3. Number of errors	0.26 **	−0.37 **	--				
4. Error RT	0.30 **	−0.29 **	−0.01	--			
5. Correct RT	0.31 **	−0.10	−0.32 **	0.77 **	--		
6. PES	−0.02	0.02	−0.32 **	0.19	0.25 *	--	
7. PEA	−0.31 **	0.40 **	−0.85 **	−0.26 **	0.06	0.38 **	--

**Note:** RT, reaction time; PES, post-error slowing; PEA, post-error accuracy; CRN, correct-related negativity; ERN, error-related negativity; Pe, post-error positivity; difference, error minus correct. Statistical significance is determined by asterisk (* *p* < 0.05, ** *p* < 0.01).

**Table 4:** Bivariate correlations among trait mindful awareness and ERP measures by group.

**Control**	**1.**	**2.**	**3.**	**4.**	**5.**	**6.**	**7.**	**8.**
1. Δ ERN	--							
2. Δ Pe	−0.19	--						
3. FFMQ Overall	−0.02	0.01	--					
4. FFMQ-O	0.04	0.10	0.42 **	--				
5. FFMQ-D	0.02	−0.11	0.75 **	0.27 **	--			
6. FFMQ-AA	−0.02	−0.05	0.64 **	−0.08	0.39 **	--		
7. FFMQ-NJ	−0.04	0.02	0.68 **	−0.08	0.33 **	0.50 **	--	
8. FFMQ-NR	−0.05	0.07	0.51 **	0.35 **	0.32 **	0.00	0.08	--
**Meditation**	**1.**	**2.**	**3.**	**4.**	**5.**	**6.**	**7.**	**8.**
1. Δ ERN	--							
2. Δ Pe	−0.20 *	--						
3. FFMQ Overall	0.05	0.05	--					
4. FFMQ-O	0.15	0.09	0.46 **	--				
5. FFMQ-D	0.15	−0.08	0.70 **	0.37 *	--			
6. FFMQ-AA	0.00	0.01	0.74 **	0.04	0.27 **	--		
7. FFMQ-NJ	−0.10	0.03	0.63 **	−0.22 *	0.26 **	0.61 **	--	
8. FFMQ-NR	−0.03	0.14	0.56 **	0.42 **	0.23 *	0.26 **	0.07	--

**Note:** ERN, error-related negativity; Pe, post-error positivity; difference, error minus correct; FFMQ, Five Factor Mindfulness Questionnaire (high scores indicate higher levels of dispositional mindfulness, overall score computed as average of all items); FFMQ-O, Observe; FFMQ-D, Describe; FFMQ-AA, Acting with Awareness; FFMQ-NJ, Nonjudgment; FFMQ-NR, Nonreactivity. Statistical significance is determined by asterisk (* *p* < 0.05, ** *p* < 0.01).
